# Graphene nanoplatelets as nanofillers in mesoporous silicon oxycarbide polymer derived ceramics

**DOI:** 10.1038/s41598-018-36080-1

**Published:** 2018-12-05

**Authors:** Ravindran Sujith, Pawan Kumar Chauhan, Jella Gangadhar, Ankur Maheshwari

**Affiliations:** Department of Mechanical Engineering, Birla Institute of Technology and Science - Pilani, Hyderabad Campus, Jawahar Nagar, Kapra Mandal, Medchal District, 500078 Telangana India

## Abstract

Understanding the role of graphene in the thermal stability and pore morphology of polymer derived silicon oxycarbide is crucial for electrochemical energy storage and hydrogen storage applications. Here in this work, we report the synthesis of graphene nanoplatelets dispersed silicon oxycarbide ceramics by the polymer to ceramic synthesis route. Samples containing graphene and without graphene are subjected to different pyrolysis conditions and are characterized using FT-IR, XPS, Raman spectroscopy, XRD, FE-SEM, HR-TEM, and BET. The results show that the graphene dispersed in the ceramic has undergone structural distortions upon pyrolysis and resulted in the formation of nanoclusters of graphene and turbostratic graphene. The XRD results confirm that with the incorporation of higher wt.% of GNP there is resistance to crystallization even at an exceedingly high pyrolysis temperature. The pores are bimodal in nature with specific surface area ranging between 22 and 70 m^2^/g and are generated *in-situ* during the polymer to ceramic conversion. Our study confirms that upon adjusting the graphene content it is possible to tune the structure and pore morphology of the polymer derived ceramics as per the requirements.

## Introduction

In recent years there is an outburst of research in the synthesis and characterization of polymer derived ceramics (PDC) for various functional applications such as in energy storage, lithium-ion batteries, photocatalysis, thermoelectrics and drug delivery^1–6^. For many of these applications, porous PDCs are considered to be advantageous than their dense counterpart. For instance, it was demonstrated by Dibandjo *et al*. that porous silicon oxycarbide exhibited a better electrochemical response in comparison to its dense counterpart for lithium storage applications^7^. The porosity in these ceramics is inherently developed during the synthesis and by proper control of the processing parameters, it is possible to tailor the pore morphologies as per the requirements. Recent two review papers published on porous PDCs summarizes the various processing strategies to be adopted for getting the proper microstructure^8,9^. Among the different pore morphologies, hierarchical porosity seems to have gained the highest attention^9,10^. In our study, we have adopted a simple method by which tunable hierarchical porosity can be attained through the proper control of the precursor chemistry. However, the usage of these mesoporous ceramics for energy storage applications such as in lithium-ion batteries is limited due to its low electrical conductivity of 3 × 10^−9^ S/cm^11^.

In this context, further enhancement of functional properties in PDCs can be achieved by the addition of conductive nano-fillers such as carbon nanotubes, graphene oxide and graphene nanoplatelets (GNP). Recent studies have shown that the addition of nanofillers like multi-walled carbon nanotubes and graphene oxide dispersoids could lead to an enhancement of electrical conductivity by three to four orders of magnitude in these ceramics^11–15^. In one such study by David *et al*. graphene oxide – silicon oxycarbide PDC system was explored as a suitable anode material for lithium-ion batteries and has shown considerably high cycling efficiency with remarkable structural stability^1^. Despite the aforementioned literature, there is no significant work reported so far on the addition of GNP into the silicon oxycarbide and its effect on the structural stability and pore morphology of the PDC. GNP is one to a few atomic layer thick material and can sustain remarkable current densities along with exceptional thermal and mechanical properties^16,17^. Hence, these GNP can be envisaged as suitable nano-fillers for these ceramics. The present work, therefore, tries to establish the influence of GNP in the polymeric precursor mixture and its effect on the pyrolyzed samples, both in amorphous and crystalline forms. Through this study we have tried to demonstrate the effect of GNP in terms of the different phases in the pyrolyzed ceramic samples, nature of the graphene left after pyrolysis, its impact on the turbostratic graphene in the ceramic and finally, the resulting effect on the pore morphology of the ceramic. This study also tries to reflect on the balance between having a high specific surface area (SSA) and turbostratic graphene content in the ceramic which are essential requirements for any functional application. Moreover, it is expected to give direction towards synthesizing a suitable material for potential electrochemical energy storage applications such as in lithium-ion batteries wherein a right mixture of amorphous and crystalline material with controlled porosity and graphene is expected to yield better results.

## Results

### Material characterization

**Figure 1:**
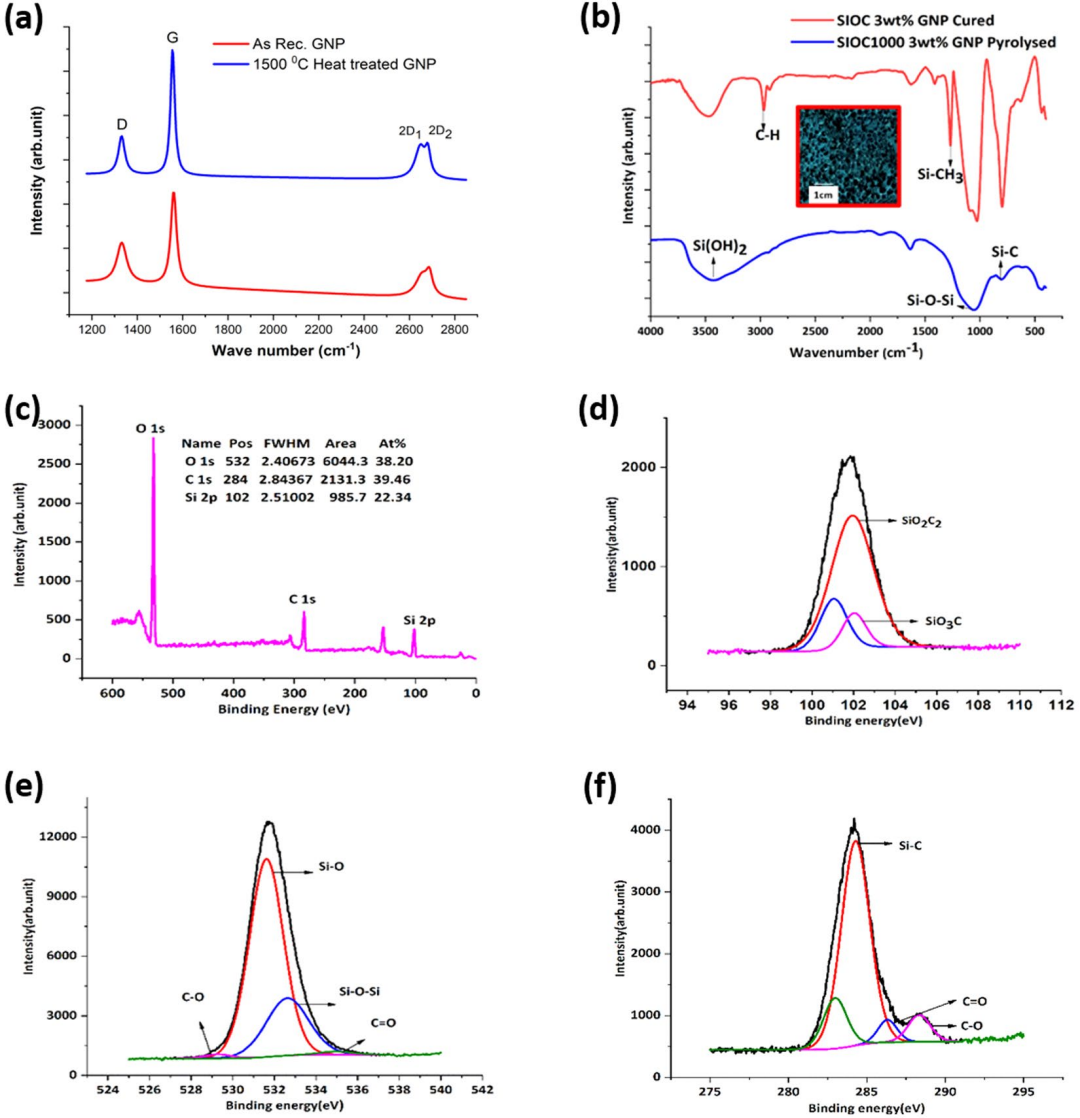
Structural evolution from polymer to ceramic. (**a**) Raman spectra of the as-received GNP and the GNP heat-treated at 1500 °C in Ar atmosphere. (**b**) Fourier Transform Infrared Spectroscopy spectra of Si-O-C after pyrolysis at 1000 °C of the 3 wt.% GNP dispersed polymeric mixture and the crosslinked GNP dispersed polymeric mixture. In the inset, the digital photograph of the pyrolyzed sample is shown and the pores can be seen. (**c**) XPS spectrum of the 3 wt.% GNP dispersed Si-O-C pyrolyzed at 1000 °C. High-resolution XPS spectrum of the pyrolyzed samples in the (**d**) C-1s region, (**e**) Si-2p region and (**f**) O-1s region. Deconvoluted peaks show the various bonds existing in the Si-O-C ceramic.

A commercially available GNP was used as the nano-filler and its structure is characterized using Raman spectroscopy and is shown in Fig. 1(a). The intense peaks observed at 1561 cm^−1^, 2655 cm^−1^ and 2688 cm^−1^ corresponds to that of G, 2D_1_ and 2D_2_ peaks of GNP and it could be inferred from their intensity ratio (I_2D_/I_G_ ~ 0.23) that it is a few layered graphene. In addition, the presence of a single sharp D (disorder induced) peak at 1332 cm^−1^ indicates that the defects are present in the GNP^18^. In order to establish the structural stability of GNP upon exposure to high temperature, these powders were subjected to heat-treatment at 1500 °C in Ar atmosphere. The Raman spectrum of the heat-treated GNP is shown in the same figure and the peaks closely match with that of the as-received GNP ensuring its structural stability (for details refer to Table 1). However, a peak shift of +1 cm^−1^ to +4 cm^−1^ was observed for all the peaks upon heat-treatment and it could be due to the change in the bond length due to the thermal stresses. Moreover, we observe sharpening of the G-peak after heat-treatment at 1500 °C (~9 cm^−1^). Further, from the Fourier transform infrared spectroscopy (FT-IR) the absence of peaks corresponding to C-H (2967 cm^−1^) and Si-CH_3_ (1268 cm^−1^) and the presence of Si-O-Si (1100 cm^−1^) and Si-C (800 cm^−1^) peaks in the pyrolyzed samples suggests the progress in polymer to ceramic conversion (Fig. 1(b)). X-ray photoelectron spectroscopy (XPS) of the 3wt.% GNP dispersed Si-O-C pyrolyzed at 1000 °C revealed the presence of O 1s, C 1s, Si 2s, Si 2p and O 2s peaks (Fig. 1 (c)). The elemental composition at the surface was found to be 39.46 at.% C (27.67 wt.%), 38.20 at.% O (35.69 wt.%) and 22.34 at.% Si (36.64 wt.%). For gaining detailed information on the local environment detailed scans were performed across each of these peaks (O 1s, C 1s and Si 2p). The O/Si atomic ratio of 1.7 is closer to the results given by chemical analysis listed in the literature^19^. However, the C/Si atomic ratio of 1.76 is relatively high which could be due to the addition of GNP resulting in the local enrichment of the C content. The peaks were deconvoluted and the corresponding fitted peaks are shown in Fig. 1 (d–f). The Si (2p) spectrum shows a peak at 101.94 eV arising due to the formation of the SiO_2_C_2_ and at 102.025 eV presence of SiO_3_C. The presence of both of these peaks confirms the amorphous nature of the ceramic. For the C (1s) spectrum, peaks were observed at 284.28, 286.31, 288.33 eV and are assigned to Si-C (SiC_4_), C=O, and C-O, respectively. Again, the highest intensity for the Si-C peak indicates towards completion of the pyrolysis process and formation of the silicon oxycarbide. Similarly, for O (1s), the peaks at 531.61, 532.62, 529.28 and 534.94 eV correspond to Si-O, Si-O-Si, C-O, and C=O, respectively.

**Table 1:** Details of Raman Spectra and XRD for the GNP and the pyrolyzed ceramics.

Sample	D peak (cm^−1^)	β (cm^−1^)	G peak (cm^−1^)	β (cm^−1^)	2D_1_* (cm^−1^)	2D_2_* (cm^-1^)	I(D)/I(G)	I(2D_1_)/I(G)	I(2D_2_)/I(G)	L_a_ (nm)	L_D_ (nm)	SiC Crystallite size (Ǻ)
As-received GNP	1332	53	1561	31	2655	2688	0.43	0.23	0.21	45	18	—
GNP-1500	1332	34	1558	22	2656	2689	0.30	0.25	0.18	64	22	—
SiOC1500	1322	93	1582	62	2650	2901	1.51	0.10	0.10	13	10	16
SiOC1500-3wt% GNP	1327	90	1588	59	2651	2905	1.47	0.11	0.10	13	10	16
SiOC1500-6wt% GNP	1319	142	1581	78	2620	2916	1.32	0.12	0.23	15	10	19

The peak intensity ratio (I_D_/I_G_, I_2D1_/I_G_, I_2D2_/I_G_), cluster size of GNP (L_a_), point defect separation distance (L_D_) and crystallite size of β-SiC are given.

*For GNP peaks corresponds to 2D_1_ and 2D_2_, and for pyrolyzed ceramics 2D_1_ and 2D_2_ should be read as 2D and D + G.

### Structural Characterization

**Figure 2:**
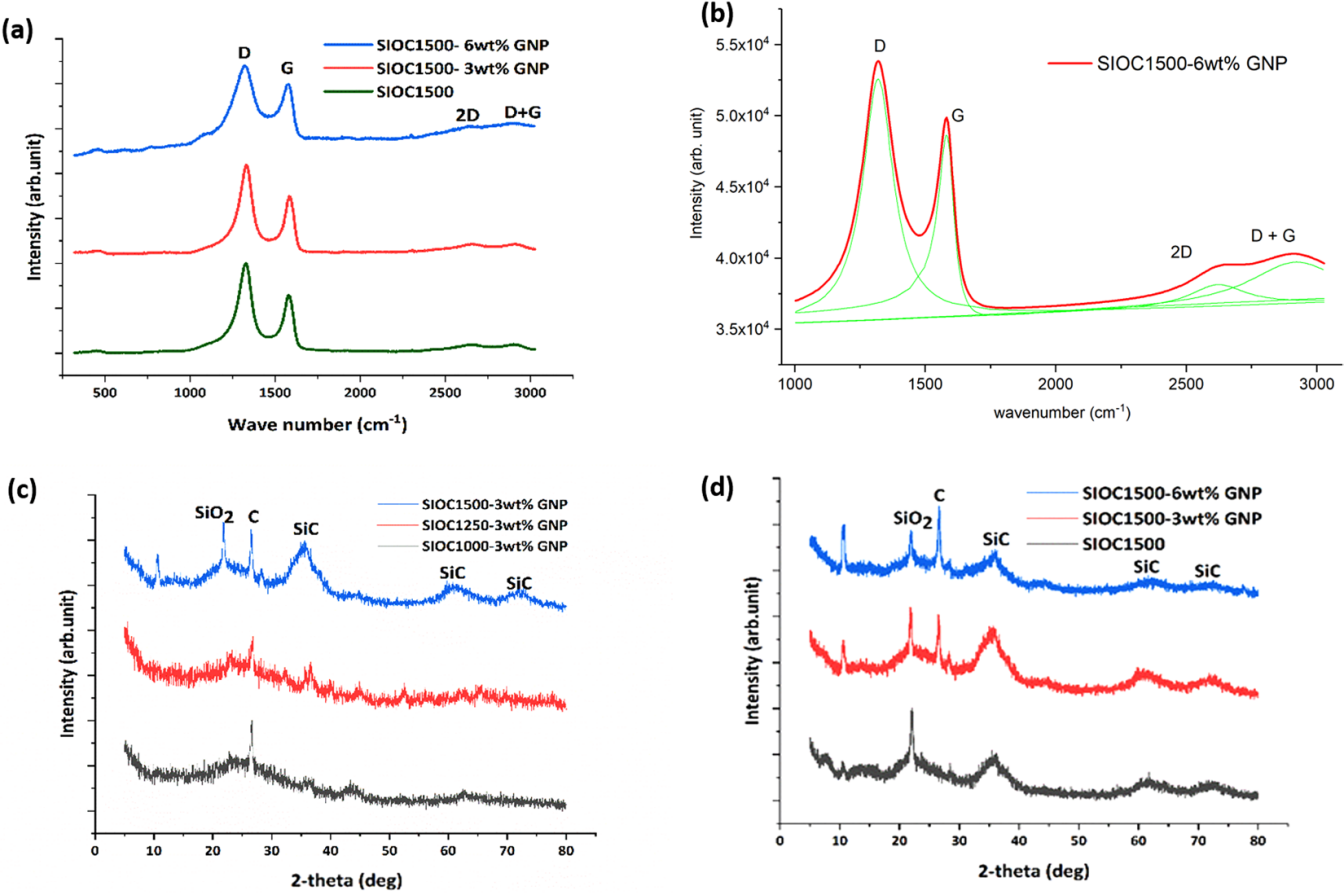
Structural characterization of pyrolyzed ceramic. (**a**) Raman spectra of Si-O-C PDC without GNP and with 3 wt.% and 6 wt.% GNP pyrolyzed at 1500 °C in Ar atmosphere. (**b**) Shows the corresponding fitted curve for the Si-O-C PDC with 6 wt.% GNP. (**c**) XRD of the Si-O-C 3 wt.% GNP pyrolyzed ceramics at 1000 °C, 1250 °C and 1500 °C is shown (**d**) XRD of the Si-O-C sample pyrolyzed at 1500 °C without GNP and with 3 wt.% and 6 wt.%, GNP is shown. All the plots shown are normalized.

Similar to GNP, the silicon oxycarbide ceramics with and without GNP pyrolyzed at 1500 °C also have peaks corresponding to D band and G band in their respective Raman spectrum (Fig. 2 (a)). However, it is to be noted that the intensity of the D peak is higher than that of the G peak for all the pyrolyzed samples inferring higher concentration of defects in comparison to GNP (Table 1). This could be associated to two reasons: (i) presence of defects in the form of grain boundaries and distortions in the GNP^20^ and (ii) presence of defects in the free carbon or turbostratic graphene produced during the polymer to ceramic conversion^16^. For further understanding, the peaks were deconvoluted (Fig. 2(b)) and the lowest I_D_/I_G_ of 1.32 was observed for the 6 wt.% GNP modified silicon oxycarbide (Table 1). A lower I_D_/I_G_ ratio reflects a better ordered graphitic structure with lower defect concentration. In addition, the 2D band (overtone of the D band) and D + G band are also present in the pyrolyzed samples. The presence of these bands and their position is strongly dependent on the structural integrity of the ceramic.

The effect of pyrolysis temperature on the structural evolution of the GNP modified ceramics can be observed from the X-ray diffractogram (XRD) shown in Fig. 2(c). The characteristic peak corresponding to carbon (2θ = 26°) can be observed in the GNP containing samples pyrolyzed at 1000, 1250 and 1500 °C. This could be corroborated with the XRD of the as-received GNP and the GNP heat-treated at 1000 °C (Supplementary Fig. 1). However, the peaks related to SiC and SiO_2_ are not so prominent in the XRD of the 1000 & 1250 °C pyrolyzed samples suggesting the predominant amorphous nature of the ceramic. In Fig. 2(d) the structural evolution of the ceramic modified with various weight fraction of GNP pyrolyzed at 1500 °C is shown. The intensity of the carbon peak seems to increase with an increase in the wt.% of the GNP. Due to phase separation, graphitic domains in the form of free carbon will be created additionally and this in presence of GNP may enhance the formation of further graphitic domains. It has been reported that the presence of graphene in the system tends to lower the crystallization activation energy of free carbon^13,15^. Further, we can observe a decrease in peak intensity corresponding to SiC peaks for 6 wt.% GNP modified silicon oxycarbide suggesting crystallization is influenced by the presence of higher carbon content in the form of GNP. For these pyrolyzed ceramics the crystallite size of the β-SiC tends to vary from ~16 Ǻ to 19 Ǻ.

**Figure 3:**
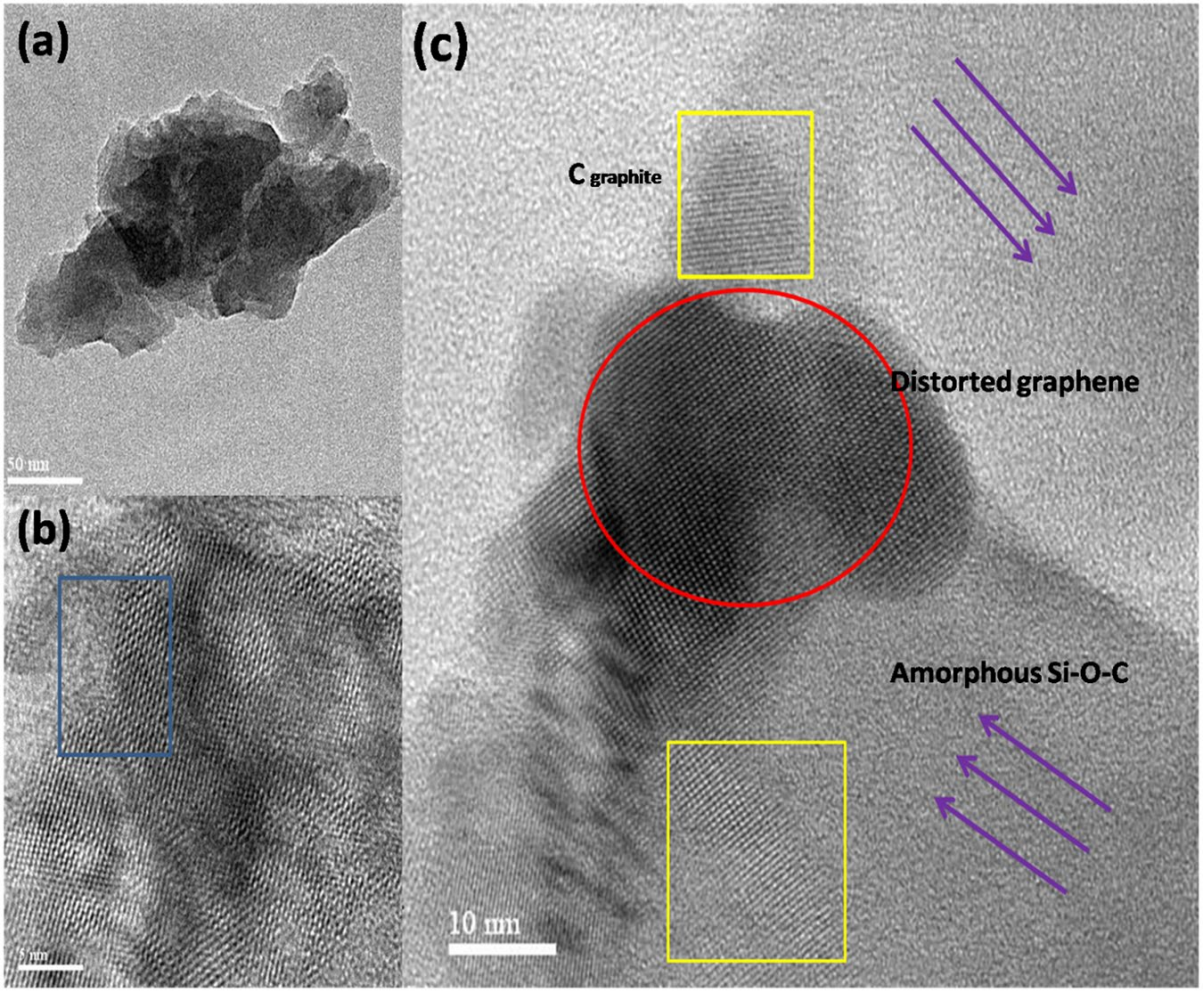
Shows the HR-TEM images of the SiOC1250 3 wt.% GNP (**a**) dark and bright regions showing GNP and Si-O-C respectively; (**b**) separation of the hexagonal graphene structure and amorphous Si-O-C phase is shown and in the inset SAD pattern is given and is a diffused spotted pattern; (**c**) shows the presence of layered GNP and turbostratic graphene dispersed in an amorphous Si-O-C matrix.

High resolution transmission electron microscope (HR-TEM) characterization was carried out to visualize the structure of GNP, free carbon and their distribution in the pyrolyzed sample. The dark areas observed in Fig. 3(a) reflect the thick stacking nanostructure of several graphene layers^1^. Further, the sample containing GNP with layers folded can be pictured in Fig. 3(b) (shown in red circle) and the folding of these layers results in the development of the Moire’s pattern. The presence of the turbostratic graphene could be observed as straight grid lines (seen within yellow rectangles) in the HR-TEM as seen in Fig. 3(c)^21^. The arrows in indigo indicate the amorphous silicon oxycarbide matrix and the rectangle in blue (Fig. 3(b)) indicates the boundary between GNP and amorphous silicon oxycarbide.

### Specific surface area characterization

**Figure 4:**
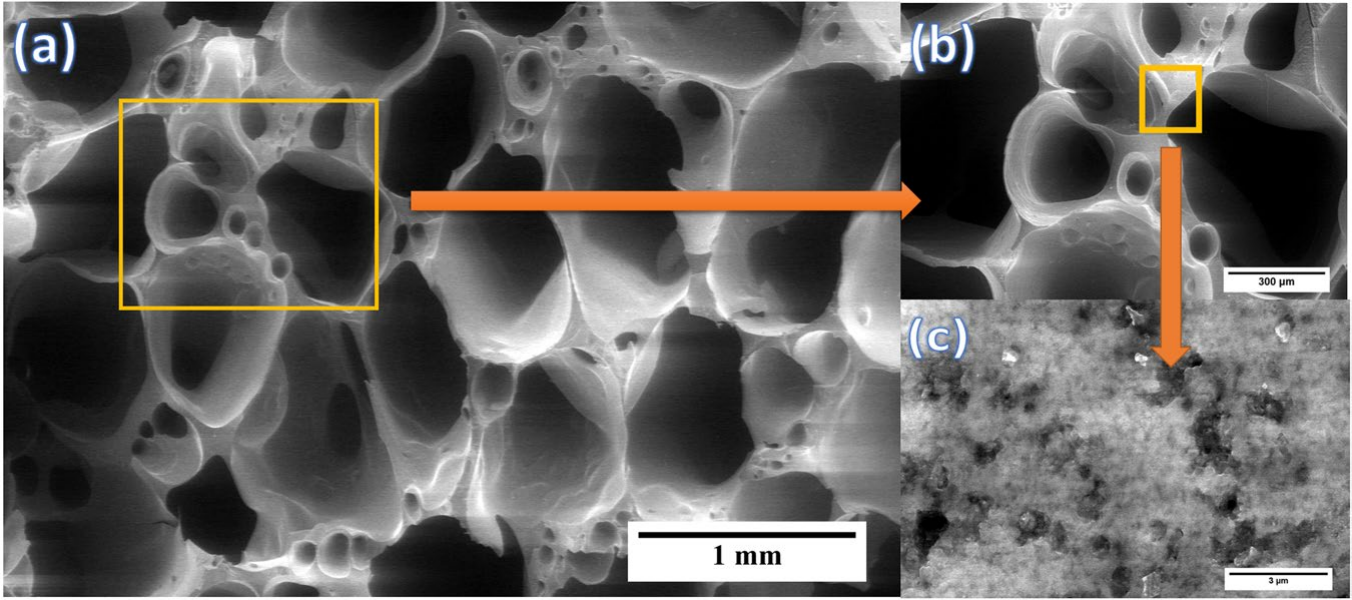
Shows the presence of bimodal porosity in the GNP modified ceramics. (**a**) zoomed out image showing the presence of micro-sized pores, (**b**) magnified image of the same indicating the micro-sized pores are aligned and (**c**) zoomed in image of the strut showing the presence of meso-sized pores.

**Figure 5:**
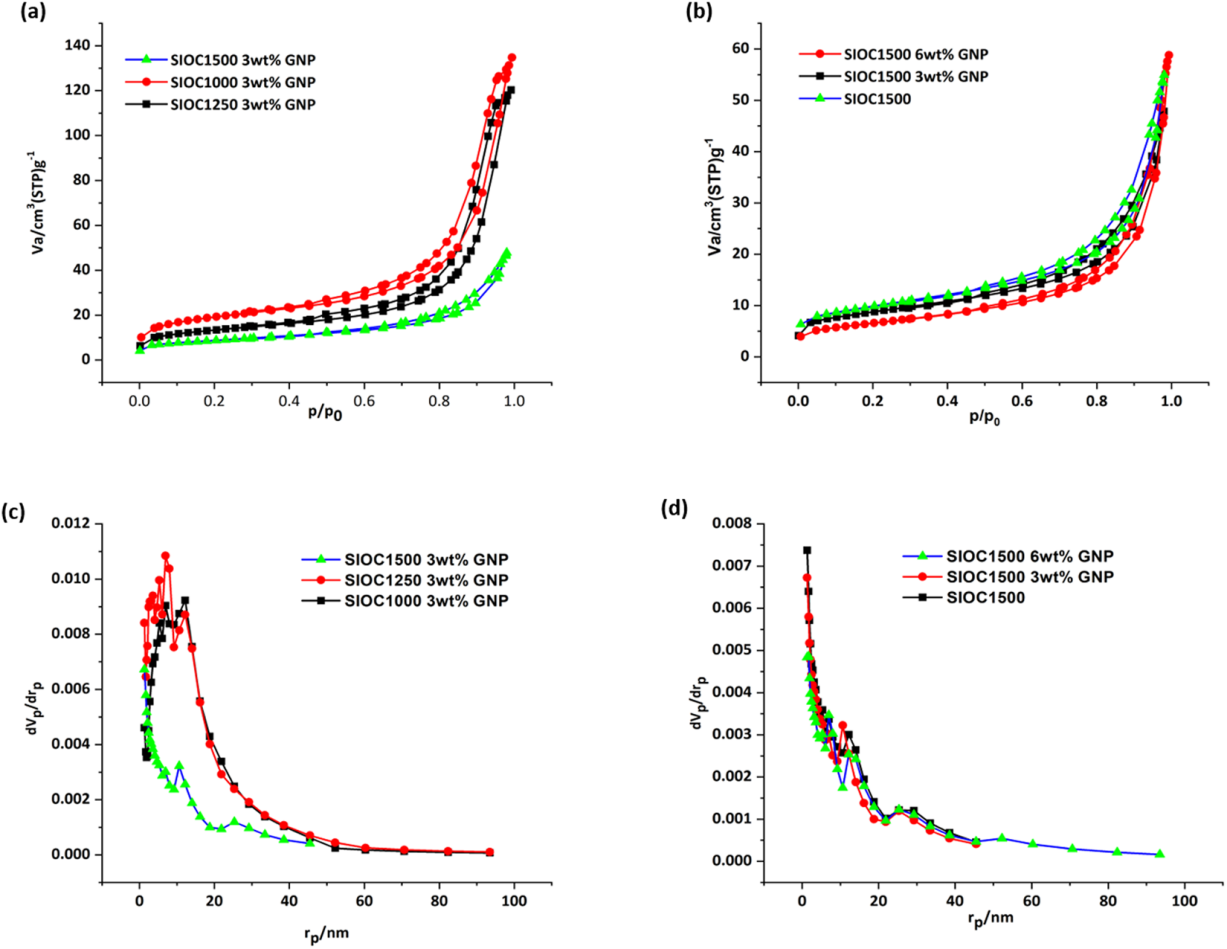
SSA and Pore size distribution: Nitrogen sorption isotherms of the samples pyrolyzed showing the hysteresis (Type IV isotherms) (**a**) at 1000, 1250 and 1500 °C, (**b**) 1500 °C sample without GNP and 3 wt.% and 6wt.% GNP. (**c**,**d**) shows the corresponding Barrett-Joyner-Halenda (BJH) analysis exhibiting the pore size distribution in the pyrolyzed samples.

The porosity in the GNP modified ceramics can be visualized from the scanning electron microscopy (SEM) images shown in Fig. 4(a). There seems to be two different size range of pores existing in these ceramics: macro-sized pores and meso-sized pores. The macro-sized pores (>50 nm) are existing as channels (Fig. 4(b)) and meso-sized pores (2 to 50 nm) are observed in the struts of the porous ceramic (Fig. 4(c)). The meso-sized pores are produced due to the escapement of the gases during the pyrolysis which gets combined together as it moves towards the surface resulting in macro-sized pores^10^. A detailed Brunauer, Emmett and Teller (BET) study further confirm this. The adsorption and desorption behavior of the samples established through BET is shown in Fig. 5(a,b). All the samples possess the hysteresis loop in the N_2_ adsorption isotherms inferring the mesoporous nature of the pores. Further, the microporosity is inferred through the coinciding adsorption and desorption curves till P/P_0_ ratios of 0.4–0.5 and the mesoporosity through the hysteresis curves beyond P/P_0_ ratios of 0.4–0.5. The results are consistent with the work carried out by Vakifahmetoglu *et al*.^10^. Our BET study also indicates an overall decrease in SSA with increase in pyrolysis temperature from 1000 to 1250 °C. However, SSA tend to be higher for the SiOC1500 sample. In case of GNP modified ceramics, SSA seem to decrease with increase in the pyrolysis temperature. In addition, samples containing GNP exhibited higher SSA compared to the samples without GNP at both 1000 °C and 1250 °C. However, the trend does not seem to hold good for the samples pyrolyzed at 1500 °C which have undergone crystallization (Table 2).

**Table 2:** Summary of BET and BJH analysis showing the distribution of SSA, pore volume and mean pore diameter for the pyrolyzed samples.

SI No.	Sample Description	SSA (m^2^/g)	Pore volume (cm^3^/g)	Mean pore diameter (nm)
1.	SiOC1000	37.50	0.17	18
2.	SiOC1250	26.60	0.15	22
3.	SiOC1500	34.49	0.08	10
4.	SiOC1000-3wt%GNP	68.25	0.20	12
5.	SiOC1250-3wt%GNP	46.94	0.18	15
6.	SiOC1500-3wt%GNP	30.54	0.07	10
3	SiOC1000-6wt%GNP	51.47	0.15	11
6	SiOC1250-6wt%GNP	44.22	0.15	14
9	SiOC1500-6wt%GNP	22.93	0.09	15

## Discussion

The Raman spectroscopy of the GNP reveals a few layered structure as seen in the results section. The G band is due to the bond stretching of sp^2^ hybridized carbon and the presence of D peak confirms the existence of defects possibly in the form of sp^3^ hybridized carbon and grain boundaries. However, after dispersing it in the polymeric mixture and subsequent pyrolysis the graphene structure revealed through Raman seems to be different. This could be associated to the presence of nanodomains of free carbon in the pyrolyzed ceramics. It has been reported in the PDC literature that pyrolysis of these C-rich ceramics can result in the formation of nanodomains of free carbon even before the phase separation process gets initiated. This free carbon could be essentially in the form of turbostratic carbon or graphene like sheets^22,23^. However, we are unable to observe the D and G peaks in the Raman spectrum for the 1000 °C and 1250 °C GNP dispersed pyrolyzed samples due to the presence of the strong background fluorescence of the amorphous ceramic^24^ [Refer to supplementary Fig. 2 for more details]. The layered carbon nanostructures dispersed in an amorphous silicon oxycarbide matrix can be seen in the HR-TEM images [Fig. 3(a–c)]. For HR-TEM studies the samples pyrolyzed at 1250 °C was chosen so as to have a clear visualization of graphene structure devoid of SiC and SiO_2_ crystallites. The selected area diffraction (SAD) pattern shown in the inset of Fig. 3(b) indicate a diffused spotted pattern and is attributed to the layered carbon^1^.

For the 1500 °C pyrolyzed samples significant overlap of the D and G bands along with the broadening of peaks was observed in the Raman spectra. This signifies that though GNP seems to be thermally stable, defects seem to have increased due to thermal distortions. Also, it could be due to the presence of a higher concentration of free carbon or turbostratic graphene domains. The cluster size of the graphene sheets (L_a_) and the point defect separation distance [L_D_] in the pyrolyzed samples is determined using the equation developed by Cancado *et al*.^25,26^ and are given by equations 1 and 2, respectively.

1 $${{\rm{L}}}_{{\rm{a}}}=2.4\times {10}^{-10}\times {\lambda }^{4}\times {(\frac{{I}_{D}}{{I}_{G}})}^{-1}$$

2 $${{\rm{L}}}_{{\rm{D}}}^{2}=1.8\times {10}^{-9}\times {\lambda }^{4}\times {(\frac{{I}_{D}}{{I}_{G}})}^{-1}$$

where λ is the excitation wavelength (532 nm) of the laser used in the Raman spectroscopy. The cluster size of the as-received GNP and GNP-1500 are 45 nm and 64 nm respectively. This indicates that ordering has increased due to the heat-treatment process. However, the cluster size of the SiOC1500 is smaller (13–15 nm), inferring the structure of the graphene is inherently different and these could be associated to the presence of free carbon or turbostratic graphene. The presence of these turbostratic graphene layers has been observed in our HR-TEM studies (Fig. 3). Hence, in our system, we have a combination of two different graphene structures: GNP and turbostratic graphene.

In the case of SiOC1500 GNP modified systems, the cluster size is found to remain same (13–15 nm) with increase in GNP weight fraction. Correspondingly, the separation distance between the defects is also found to remain same (~10 nm). A similar trend in the response of cluster size with carbon nano-fiber weight fraction is reported in the work carried out by Mazo *et al*. in the carbon fiber incorporated SiOC nanocomposites^27^. Further, these graphene nanoclusters could have reacted with SiO_2_ and formed additional SiC, thereby resulting in an overall decrease in the intensity of the SiO_2_ peak at 2θ = 22° (Fig. 3(d))^28^. This illustrates that there are two mechanisms of SiC formation in the existing system: (i) by phase separation and (ii) the phase separated SiO_2_ reacting with the existing turbostratic graphene and forming SiC. This suggests that there should be an increase in the intensity of the peak of SiC for 6wt.% GNP modified ceramic. However, we have not observed an increase in the peak intensity for SiC crystallites. We believe that the only way this could have happened is the decrease in the formation of SiC by phase separation, suggesting resistance to crystallization. There are literature reports which suggests that the presence of high volume fraction of carbon can inhibit crystallization and growth of SiC^29,30^. However, further studies are required to establish this.

The effect of GNP’s on the evolution of pores in these ceramics was studied using BET analysis. From Table 2 it could be inferred that there is an increase in pore size with increase in pyrolysis temperature till 1250 °C. Further upon pyrolysis at 1500 °C the pore size seems to reduce, indicating densification with crystallization. However, the extent of densification in the crystallized samples is decreased with the increase in GNP weight fraction. Hence the sample with 6 wt.% GNP has largest mean pore diameter and minimum SSA. In addition, studies on the pore size distribution was done using BJH analysis. Figure 5c shows the densification of the ceramic upon crystallization with most of pores in the meso-range (2 to 50 nm). Moreover, the effect of weight fraction of GNP on the pore size distribution of crystallized ceramics can be inferred from Fig. 5d. Unlike the 0 and 3 wt.% GNP incorporated system the 6 wt.% GNP ceramic has pores distributed in the macro-regime (>50 nm) correlating with the result observed in the BET analysis.

## In Summary

In the present work, various characterization techniques were used to study the effect of GNP on the structural evolution, pore size distribution, and its morphology in the silicon oxycarbide ceramics. The Raman spectroscopy studies coupled with XRD and HR-TEM confirms that there are two types of graphene structures existing in these GNP modified PDCs. Our study suggests that the phase separation and crystallization in these PDCs is hindered at higher weight fractions of GNP. Moreover, the presence of GNP seems to have a different influence on the pore size distribution for the amorphous and crystalline ceramics. In amorphous systems, the presence of GNP resulted in an increase in SSA, whereas the reverse was observed to be true in crystalline PDC. Hence, the present work gives direction towards the synthesis of GNP dispersed PDC materials which can have the appropriate combination of porosity, pore size, SSA and balance of amorphous and crystalline phases as per the requirement.

## Methods

### Chemicals

To synthesize silicon oxycarbide, three polymeric precursors were chosen: a linear polymethylhydrosiloxane [PHMS, (CH_3_)_3_SiO{(CH_3_)HSiO}nSi(CH_3_)_3_, viscosity: 15–40 mPa.s (20 °C), CAS Number 63148-57-2, Sigma Aldrich, USA] for getting the basic structure of ceramic, vinyl terminated polydimethylsiloxane [PDMS,CH_2_CH{Si(CH)_2_O}nSi(CH_3_)_2_ CHCH_2_, viscosity:850–1150 mPa.s (25 °C), CAS Number 68083-19-2, Sigma Aldrich, USA] as a sacrificial pore former, cyclic 2,4,6,8-tetramethyl-2,4,6,8 tetravinlycyclotetrasiloxane [TMTVS, C_12_H_28_O_4_Si_4_, CAS Number 2554-06-05, Sigma Aldrich, USA) with SiC=C moieties] for increasing the crosslinking density. Hydrotalcite [LDH with formula {Mg_6_Al_2_(CO_3_) (OH)_16_·4H_2_O}, CAS Number 11097-59-9, Sigma-Aldrich, St. Louis, MO, USA], was used in the as-received condition for aligning the pores. Platinum (0)-1,3-divinyl-1,1,3,3-tetramethyldisiloxane complex solution in xylene, Pt2%, [O{Si(CH_3_)_2_CH=CH_2_}_2_ Pt, Sigma Aldrich, USA] was used as the catalyst for the crosslinking reactions. Graphene (CAS NO.: 7782-42-5, United Nanotech Innovations Pvt. Ltd., Bangalore, India) with the specific surface area (SSA) of 300 m^2^/g was taken for dispersing in the polymeric precursor mixture. All materials are used without further purification.

### Preparation of graphene dispersed silicon oxycarbide ceramic

For comparative study three compositional combinations were considered, one was taken without the addition of graphene and the other two with graphene nanoplatelets, 3 wt.% and 6wt.%, respectively. For the three compositional combinations, the weights of the precursors were taken as, PHMS (5 g), PDMS (3.75 g) and TMTVS (2.5 g) along with LDH (2.5 g). The detailed synthesis procedure of Si-O-C PDC is as mentioned in^31^. In all the cases, the blends were put under magnetic stirring for 10 min at 500 rpm for attaining a homogenous mixture of graphene dispersed polymeric precursor. Subsequently, 100 ppm by weight of Pt relative to PHMS was added into the homogenized blends. The blends were further stirred for 30 min at 500 rpm and then were transferred to a ceramic mold. The ceramic mold was then kept on a hot plate maintained at 230 °C for 8 h for crosslinking. Once crosslinking is done, pyrolysis of these samples was carried out at 1000, 1250 and 1500 °C, respectively in a tubular furnace at a heating rate of 4 °C/min in an atmosphere of argon, followed by a dwell time of 1 h.

### Instrumentation and Characterization

The completion of polymer to ceramic conversion was established through Fourier transform- infrared spectroscopy (FT-IR) (JASCO FT-IR 4200, Japan). The FTIR analysis was carried out on the samples pyrolyzed at 1000 °C. X-ray photoelectron spectroscopy (XPS) capable of depth profiling by Ar etching was carried out using Omicron Nanotechnology (UK) to determine the surface elemental composition and local environment. Raman spectroscopy was carried out using UniRAM micro-Raman mapping system (Japan) with a laser of wavelength 532 nm. Data acquisition was carried out for an exposure time of 30 s. Ferrari and Robertson have suggested an alternate Raman spectrum fitting method using Breit–Wigner–Fano (BWF) profile for G Peak and Lorentzian for D, 2D and D + G Peak. The position of G Peak is shifted in this method and is calculated using equation ${\omega }_{max}={\omega }_{O}+\frac{(FWHM)}{2\,Q}$ where, Q^−1^ is the BWF coupling coefficient^32^. X-ray diffraction (XRD) was carried out using Rigaku - Ultima IV (Japan) with Cu: Kα (λ = 0.154 nm) to establish the effect of graphene on the various phases formed in pyrolyzed samples, conformance of the crystallization and effect of graphene on crystallization of the PDC. All the peaks were fitted using Voigt function and the crystallite sizes were determined using the Scherrer equation from the full width half maximum (β). High-resolution transmission electron microscopy (HR-TEM) was done using JEOL 3010 (USA) with a UHR pole piece operating at an accelerating voltage 300 kV. The samples were pulverized for the HR-TEM analysis. Field emission – Scanning Electron Microscope (FE-SEM) was carried out using FEI ApreoS (USA) at low vacuum mode and 50 Pa pressure. All the samples were polished following the standard metallurgical practice and ultrasonicated before carrying out FE-SEM. The images were taken using a secondary electron detector. Brunauer, Emmett and Teller (BET, BESORP-mini II, Japan) analysis was used for determining the SSA and Barrett-Joyner-Halenda (BJH) analysis for the determination of pore size distribution in the pyrolyzed samples. Type IV isotherms are used for the analysis of BJH plots. All the samples were pulverized before the BET and BJH analysis.

## Electronic supplementary material

Supplementary Figures
