# Supplementary material for: Graphene nanoplatelets as nanofillers in mesoporous silicon oxycarbide polymer derived ceramics
Source: Sci Rep. 2018 Dec 5;8:17633. doi: 10.1038/s41598-018-36080-1 (PMC6281675; doi:10.1038/s41598-018-36080-1)
Supplement: Supplementary file 1 — Supplementary Figures [file 41598_2018_36080_MOESM1_ESM.docx]

**Graphene nanoplatelets as nanofillers in mesoporous silicon oxycarbide polymer derived ceramics.**

Ravindran Sujith^*^, Pawan Kumar Chauhan, Jella Gangadhar & Ankur Maheshwari

*Department of Mechanical Engineering, Birla Institute of Technology and Science Pilani Hyderabad Campus, Hyderabad -500078 (India).*

*sujith@hyderabad.bits-pilani.ac.in





**Supplementary Figure 1:** XRD of the as-received GNP and GNP heat-treated at 1000̊C is shown. The sharp peak at 2θ = 26̊ is attribute to carbon.





**Supplementary Figure 2:** Raman spectra of Si-O-C dispersed with 3 wt% GNP pyrolyzed at 1000, 1250 and 1500̊C are shown. The peaks are not visible for the 1000 and 1250̊C pyrolyzed samples due to the high background fluorescence.
